# Characteristics of canine oral tumors: Insights into prevalence, types, and lesion distribution

**DOI:** 10.5455/javar.2023.j709

**Published:** 2023-09-30

**Authors:** Chakkarin Satthathum, Supreeya Srisampane, Pollawat Jariyarangsrirattana, Pitak Anusorn, Panpicha Sattasathuchana, Naris Thengchaisri

**Affiliations:** 1 Faculty of Veterinary Medicine, Surgery Unit, Kasetsart University Veterinary Teaching Hospital, Kasetsart University, Bangkok, Thailand; 2 Faculty of Veterinary Medicine, Veterinary Diagnostic Center, Kasetsart University, Bangkok, Thailand; 3 Department of Companion Animal Clinical Sciences, Faculty of Veterinary Medicine, Kasetsart University, Bangkok, Thailand

**Keywords:** Acanthomatous ameloblastoma, fibrosarcoma, melanoma, oral tumors, osteosarcoma, squamous cell carcinoma

## Abstract

**Objective:**

The escalating prevalence of canine oral tumors has emerged as a considerable health concern. This study examined the prevalence, types, and distributions of lesions linked to canine oral tumors.

**Material and Methods:**

The medical records of 526 dogs diagnosed with oral tumors were analyzed to determine the prevalence, types, and distributions. Tumor stages were classified into four categories using the tumor node metastasis system.

**Results:**

Among the 526 dogs, there were 118 cases of benign tumors and 408 cases of malignant tumors. Acanthomatous ameloblastoma was the most common benign tumor (43.22%), while melanoma was the most common malignant tumor (51.23%). The gingiva was the most common site for both benign and malignant lesions, accounting for 89.83% and 63.73% of cases, respectively. Melanoma, squamous cell carcinoma, and fibrosarcoma were primarily located in the gingiva, whereas osteosarcoma was commonly found in the mandible. Most tumors were classified as stage III (ranging from 46.84% to 74.58%). Of the reported cases, 56.08% were males and 43.92% were females, and the most common breed was mixed at 30.41%, followed by Poodle at 14.25% and Shih Tzu at 11.40%. Moreover, patients with malignant oral tumors (11.6 ± 3.1 years) were significantly older than those with benign tumors (8.9 ± 3.4 years,* p* < 0.0001).

**Conclusion:**

Gingiva was the primary site for oral tumors, and mainly classified as stage III. These findings emphasize the increasing occurrence of oral tumors in senior and geriatric dogs and provide insights into the prevalent types and distribution.

## Introduction

Canine malignant oral tumors make up approximately 6%–7% of all canine malignant tumors, with the oral cavity identified as the fourth most common location [1–3]. Chronic inflammation, mechanical injury, or drug administration can lead to the development of neoplastic and tumor-like lesions. In both dogs and cats, oral neoplasia is responsible for a small percentage of all cancers. Among dogs, melanoma, squamous cell carcinoma (SCC), and fibrosarcoma are the most prevalent oral malignancies. On the other hand, in cats, SCC and fibrosarcoma are frequently observed [4]. Standard treatment typically involves a wide, extensive surgical resection to remove the tumor, with the addition of chemotherapy, immunotherapy, and radiation in cases of incomplete tumor removal or high-metastasis tumors [5].

In veterinary medicine, the exact localization and prognosis of most oral lesions are not well documented. Multiple epidemiological investigations have been conducted on oral tumors in dogs and cats [1–3,4,6]. In human medicine, it is estimated that 50% of oral cancers develop from precursor lesions, highlighting the significance of early detection and proper management of pre‐malignant lesions in preventive programs [7]. Various cancer types encountered in veterinary clinics demonstrate exceptional similarities to their human counterparts in terms of genetics, molecular characteristics, and clinical presentation [8]. This underscores the immense potential of utilizing companion animals in research to advance our understanding and treatment of cancer in both human and veterinary medicine. Moreover, the histopathologic features of canine oral neoplasia in Thailand have not been described previously. Therefore, investigating the prevalence of canine oral tumors and identifying the risk factors associated with developing certain types of canine oral neoplasia [7] would not only be helpful in guiding veterinarians to perform appropriate diagnostic tests but also in providing information to develop strategies aimed at reducing morbidity and mortality associated with oral neoplasia.

The primary aim of this study was to determine the incidence and distribution of canine oral tumors. Additionally, we conducted an analysis of age, breed, and gender characteristics among dogs afflicted with oral tumors. Furthermore, the investigation explored the anatomical distribution of different oral tumors and their staging based on the TNM classification system.

## Materials and Methods

### Ethical approval

This study received approval from both the Kasetsart University Institutional Animal Care and Use Committee (approval number #ACKU61-VET-065) and the Ethical Review Board of the Office of the National Research Council of Thailand (NRCT license U1-07457-2561). Written consent was obtained from all dog owners, and the study strictly adhered to the animal care and use standards set by Kasetsart University.

### Study period and location

A retrospective review was conducted on cases of canine oral tumors that were presented at the Kasetsart University Veterinary Teaching Hospital, Faculty of Veterinary Medicine, spanning from January 2017 to December 2022.

### Study samples

The information was collected from dogs that had been diagnosed with oral neoplasia in the database of the Kasetsart University Veterinary Teaching Hospital Medical Record. Complete medical records and histological slides were evaluated.

Information gathered from the medical records included age, breed, sex, weight, oral cavity lesion, tumor classification, and clinical tumor staging. Histological slides were prepared using formalin-fixed and paraffin-embedded tissues obtained from surgical biopsies. The categorization of all oral lesions was performed according to the adjusted World Health Organization (WHO) classification. Breeds were grouped into categories based on weight, as follows: small breed: ≤12 kg; medium breed: 12–24 kg; and large breed: >24 kg.

The tumor stages were classified into four categories using the WHO tumor node metastasis (TNM) staging system. Stage I denotes a tumor size of less than 2 cm in diameter, while stage II corresponds to a tumor ranging between 2 and 4 cm in diameter. Stage III indicates a tumor with a diameter exceeding 4 cm, with or without metastasis in the lymph nodes, and stage IV represents a tumor with either lymph node involvement or distant metastasis.

### Statistical analysis

The statistical analysis of canine oral tumors was performed using STATA12 (StataCorp, College Station, TX). The data are presented as percentages, and the association between different categorical parameters was evaluated using Fisher’s exact test. The distribution of neoplastic lesions in 11 locations of the canine oral cavity was compared between benign and malignant cases using the student’s *t*-test. A significance level of *p* < 0.05 was considered statistically significant.

## Results

**Figure 1.**
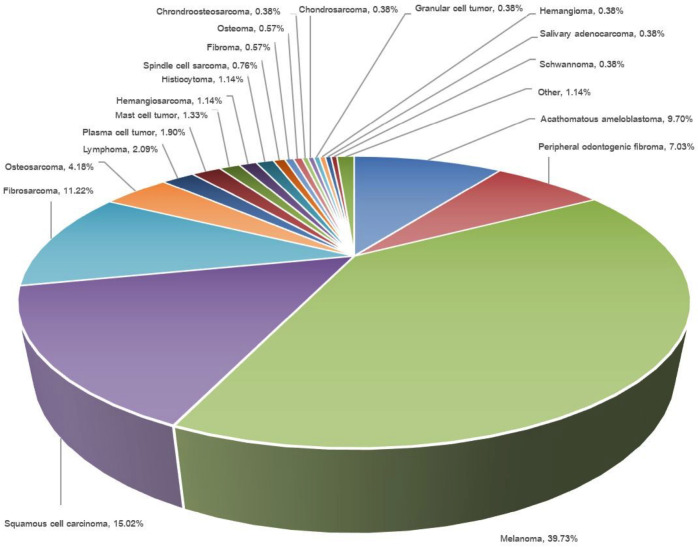
Distribution of canine oral tumors.

The total number of reported cases was 526, which included 118 dogs with benign tumors and 408 dogs with malignant tumors (Table 1). Among the reported cases, melanoma was the most prevalent tumor, constituting 39.73% of the total, while SCC followed closely, accounting for 15.02% of the cases (Figure 1). Acanthomatous ameloblastoma and fibrosarcoma were also found in 9.70% and 11.22% of cases, respectively. The top five tumor types (melanoma, SCC, fibrosarcoma, acanthomatous ameloblastoma, and osteosarcoma) accounted for approximately 81% of all cases. The remaining types of tumors were less common, with each representing less than 5% of cases (Figure 1). The mean age of the dogs with malignant tumors (11.6 ± 3.1 years) was significantly higher than that of patients with benign tumors (8.9 ± 3.4 years,* p* < 0.0001). Most cases were small breeds, and there was not a notable variation in the breed distribution among the benign and malignant groups. The mean body weight of the patients with benign tumors (16.2 ± 13.1 kg) was slightly higher than that of patients with malignant tumors (14.1 ± 10.4 kg), but the difference was not statistically significant (*p* = 0.651) (Table 1).

Of the breed types, mixed breed was the most common with 160 cases, of which 63.12% were male and 36.88% were female (Table 2). Poodle was the second most reported breed with 75 cases, of which 42.67% were male and 57.33% were female. Shih Tzu was the third most reported breed with 60 cases, of which 63.33% were male and 36.67% were female (Table 2). Most breeds had a higher percentage of males affected by oral tumors than females, except for the Poodle, Golden Retriever, Chihuahua, Beagle, and Yorkshire Terrier. There were a total of 295 male dogs (56.08%) and 231 female dogs (43.92%).

**Table 1.** Characteristics of canine patients with oral tumors.

Category	Subtype	Tumor type		*p*-value
		Benign	Malignant	
Number	**–**	118	408	**–**
Age (years)	**–**	8.9 ± 3.4	11.6 ± 3.1	<0.0001
Sex, no. (%)	Male	67 (56.78)	228 (55.88)	**–**
	Female	51 (43.22)	180 (44.12 )	0.916
Breeds, no. (%)	Small	66 (55.93)	245 (60.05)	**–**
	Medium	28 (23.73)	94 (23.04)	**–**
	Large	24 (20.34	69 (16.91)	0.613
Body weight (kg)	**–**	16.2 ± 13.1	14.1 ± 10.4	0.0651

The most common type of benign tumor in canine patients was acanthomatous ameloblastoma, representing 43.22% of all benign tumors. Peripheral odontogenic fibroma was the second most frequent type, constituting 31.31% of cases. All other types of benign tumors were relatively uncommon, with each type representing less than 5% of cases (Table 3). Of the 408 cases of malignant tumors, melanoma was the most common, representing 51.23%, followed by SCC at 19.36% and fibrosarcoma at 14.46% (Table 4). Other malignant tumor types were relatively rare, each accounting for less than 5% of cases (Table 4).

**Table 2.** Breed distribution among dogs with oral tumors.

Breed	Male, no. (%)	Female, no. (%)	Total no. (%)
Mixed	101 (63.12)	59 (36.88)	160 (30.41)
Poodle	32 (42.67)	43 (57.33)	75 (14.25)
Shih Tzu	38 (63.33)	22 (36.67)	60 (11.40)
Golden Retriever	16 (44.44)	20 (55.56)	36 (6.84)
Pomeranian	13 (54.17)	11 (45.83)	24 (4.56)
Chihuahua	8 (42.11)	11 (57.89)	19 (3.61)
Labrador Retriever	11 (64.71)	6 (35.29)	17 (3.23)
Beagle	7 (43.75)	9 (56.25)	16 (3.04)
Bangkaew	8 (66.67)	4 (33.33)	12 (2.28)
Jack Russell Terrier	7 (63.64)	4 (36.36)	11 (2.09)
French Bulldog	7 (70.00)	3 (30.00)	10 (1.90)
Yorkshire Terrier	4 (40.00)	6 (60.00)	10 (1.90)
Others	43 (56.58)	33 (43.42)	76 (14.44)
Total	295 (56.08)	231 (43.92)	526 (100)

In both benign and malignant lesions, the gingiva was the predominant site, constituting 89.83% of benign lesions and 63.73% of malignant lesions (Table 5). The tongue was the second-most frequent location for benign lesions, constituting 5.08% of cases. Other sites where benign lesions were observed include the tonsil (0.85%), lip (1.69%), and mandible (2.54%) (Table 5). The lip was the second-most common site for malignant lesions, representing 7.84% of cases. Other sites where malignant lesions were observed include the buccal mucosa (6.62%), hard palate (4.41%), tongue (4.41%), tonsil (3.92%), soft palate (2.94%), mandible (3.68%), and maxilla (1.96%) (Table 5).

Among the 209 cases of melanoma, 69.86% were located in the gingiva, followed by 8.13% on the lips and 7.66% on the buccal mucosa (Table 6). For SCC, 74.68% of the 59 cases were located in the gingiva, and 10.13% were located in the tonsils. No cases of melanoma or SCC were found in the mandible or maxilla (Table 6). The highest percentage of fibrosarcoma cases occurred in the gingiva (66.10%), followed by the hard palate (16.95%), lip (10.17%), and buccal mucosa (6.78%) (Table 6). Most osteosarcoma cases occurred in the mandible (62.50%), followed by the maxilla (25.00%) and the hard palate (4.17%) (Table 6). Conversely, acanthomatous ameloblastoma was solely identified in the gingiva, accounting for 100% of the 51 cases (Table 6).

**Figure 2.**
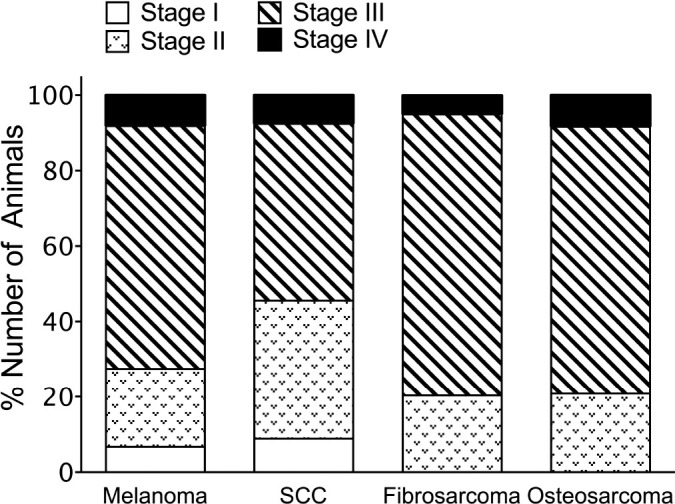
Oral tumor staging in canine malignant oral tumors according to the WHO TNM system. SCC, squamous cell carcinoma.

Most cases for all tumor types were classified as WHO TNM stage III, ranging from 46.84% for SCC to 74.58% for fibrosarcoma (Figure 2). The percentage of stage I and stage IV cases for all tumor types was relatively low, ranging from 0% to 8.17% (Figure 2).

## Discussion

The present study revealed a notable age difference between dogs with malignant tumors and those with benign tumors, indicating that dogs with malignant tumors were significantly older. This finding is consistent with previous research that suggested an age-related increase in oral tumor incidence [4–6]. However, there was no noticeable variation in breed distribution between the groups of dogs with benign versus malignant tumors. Contrary to previous research indicating that overweight dogs are more prone to developing specific types of tumors [9,10], no significant distinction in the average body weight of dogs with benign and malignant tumors was observed. However, the difference in body weight between the two groups identified in previous studies was small and may not be clinically significant.

The present retrospective study showed that most oral tumors in dogs were malignant, consistent with the findings of another recent study [4]. The most common types of malignant oral tumors in this study, in decreasing order, were melanoma, SCC, and fibrosarcoma. Canine oral melanoma is recognized as the most prevalent form of oral tumor [7,11], with most cases presenting as a brownish-black, firm to friable, and ulcerated mass. However, some cases of amelanotic melanoma may be pinkish [12]. In previous studies, Scottish Terriers, Golden Retrievers, Poodles, Dachshunds, Cocker Spaniels, and Chow Chows have been found to be overrepresented breeds among dogs with oral melanoma [13]. In the present study, the most common oral melanoma was diagnosed in mixed breeds: Poodles, Shih Tzus, and Golden Retrievers. Oral melanoma was most often located in the gingiva, followed by the lip and buccal mucosa, which is consistent with previous research [12]. A male predisposition to oral melanoma was observed in this study, which is also similar to previous findings [14]. The average age of dogs with canine oral melanoma in one presented study was about 12 years [15], while a previously published study reported an average age of 11 years [13]. Canine oral malignant melanoma is an aggressive tumor with a poor prognosis. Early surgical removal is essential for long-term survival in stage I cases (<2 cm diameter tumor) [16], but advanced cases often experience metastases, treated with radiotherapy and chemotherapy [17].

**Table 3.** Distribution of benign canine oral tumor types.

Tumor type	No.	% (95% CI)
Acanthomatous ameloblastoma	51	43.22 (34.13–52.66)
Fibroma	3	2.54 (0.53–7.25)
Granulosa cell tumor	2	1.69 (0.21–6.99)
Hemangioma	2	1.69 (0.21–6.99)
Histiocytoma	6	5.08 (1.89–10.74)
Lymphangioma	1	0.85 (0.02–4.63)
Papilloma	1	0.85 (0.02–4.63)
Osteoma	3	2.54 (0.53–7.25)
Peripheral odontogenic fibroma	37	31.31 (23.13–40.54)
Plasma cell tumor	10	8.47 (4.14–15.03)
Schwannoma	2	1.69 (0.21–6.99)
Total	118	100

CI, confidence interval.

**Table 4.** Distribution of malignant canine oral tumor types.

Tumor type	No.	% (95% CI)
Osteosarcoma	22	5.39 (3.41–8.05)
Basal cell carcinoma	1	0.25 (0.01–1.36)
Chondroosteosarcoma	2	0.49 (0.06–1.76)
Chondrosarcoma	2	0.49 (0.06–1.76)
Fibrosarcoma	59	14.46 (11.19–18.25)
Hemangiosarcoma	6	1.47 (0.54–3.17)
Liposarcoma	1	0.25 (0.01–1.36)
Lymphoma	11	2.70 (1.35–4.77)
Mast cell tumor	7	1.72 (0.69–3.50)
Melanoma	209	51.23 (46.26–56.17)
Rhabdomyosarcoma	1	0.25 (0.01–1.36)
Squamous cell carcinoma	79	19.36 (15.64–23.54)
Salivary adenocarcinoma	2	0.49 (0.06–1.76)
Spindle cell sarcoma	4	0.98 (0.27–2.49)
Transmissible venereal tumor	1	0.25 (0.01–1.36)
Undifferentiated carcinoma	1	0.25 (0.01–1.36)
Total	408	100.00

CI, confidence interval.

**Table 5.** Site of lesions classified as benign or malignant.

Site	Benign, no.	% (95% CI)	Malignant, no.	% (95% CI)
Gingiva	106	89.83 (82.91–94.63)	260	63.73 (58.85–68.40)
Lip	2	1.69 (0.21–5.99)	32	7.84 (5.43–10.89)
Tongue	6	5.08 (1.89–10.74)	18	4.41 (2.64–6.88)
Tonsil	1	0.85 (0.02–4.63)	16	3.92 (2.26–6.29)
Mandible	3	2.54 (0.53–7.25)	15	3.68 (2.07–5.99)
Maxilla	–		8	1.96 (0.85–3.83)
Buccal mucosa	–		27	6.62 (4.41–9.48)
Hard palate	–		18	4.41 (2.64–6.88)
Lingual	–		2	0.49 (0.06–1.76)
Soft palate	–		12	2.94 (1.53–5.08)
Total	118	100.00	408	100.00

CI, confidence interval.

**Table 6.** Site of lesions of melanoma, SCC, fibrosarcoma, osteosarcoma, and acanthomatous ameloblastoma, no. (%).

Sites	Melanoma	SCC	Fibrosarcoma	Osteosarcoma	Acanthomatous ameloblastoma
Gingiva	146 (69.86)	59 (74.68)	39 (66.10)	2 (8.33)	51 (100)
Lip	17 (8.13)	4 (5.06)	6 (10.17)	–	–
Tongue	5 (2.39)	3 (3.80)	–	–	–
Tonsil	8 (3.83)	8 (10.13)	–	–	–
Mandible	–	–	–	15 (62.50)	–
Maxilla	–	–	–	6 (25.00)	–
Buccal mucosa	16 (7.66)	4 (5.06)	4 (6.78)	-	–
Hard palate	6 (2.87)	1 (1.27)	10 (16.95)	1 (4.17)	–
Soft palate	11 (5.26)	–	–	–	–
Lingual	–	–	–	–	–
Total	209	79	59	24	51

SCC, squamous cell carcinoma.

In this study, SCC emerged as the second most commonly identified oral tumor. Consistent with previous research, SCC has been recognized as a prevalent type of malignant oral tumor in dogs [18,19]. SCC is an epithelial cell tumor that is often a bone-invasive and locally aggressive tumor. The diagnosis of SCC has predominantly been reported in medium- to large-breed dogs [20], which contrasts with the present finding. Moreover, mixed breeds, Shih Tzus, and Poodles were overrepresented in the present study. Oral SCC in dogs is classified as either tonsillar or nontonsillar [1]. The present study revealed that nontonsillar oral SCC was more prevalent, accounting for approximately 90% of the cases. In other studies, nontonsillar oral SCC constituted approximately 50%–78% of cases [21]. Oral SCC has been reported to affect senior and geriatric dogs, with a median age ranging from 8 to 10 years [22,23]. In the present study, the median age of dogs diagnosed with oral SCC was about 11 years, with a higher occurrence in males than females. The general treatment protocol for patients with oral SCC involves either chemoradiation or aggressive surgical removal of the tumor, followed by radiation therapy (RT) with or without chemotherapy [24]. Interestingly, a recent study has indicated that combining RT with immunotherapy may lead to improved response rates in dogs, similar to findings observed in humans [25]. In human research, investigations have been conducted on the use of anti-CTLA-4 immunotherapy for head and neck SCC, either as a monotherapy or in combination with other immune-checkpoint inhibitors (anti-programmed cell death protein 1, anti-programmed cell death ligand 1) or radiotherapy [26]. A comprehensive analysis of immunotherapeutic targets in dogs indicates their potential translational significance for forthcoming comparative radio-immunotherapy trials in the future.

Fibrosarcoma was identified as the third-most common oral tumor. It is characterized by malignant mesenchymal cells that are locally invasive and infiltrate adjacent bone. Fibrosarcoma in dogs presents as solid, pink-to-red lumps, commonly affecting the maxillary gingiva and the hard and soft palates in the oral cavity [27]. In this study, the most common location for oral fibrosarcoma was the gingiva, followed by the hard palate, lip, and buccal mucosa, with a median age at diagnosis of approximately 11 years. According to a previous study, oral fibrosarcoma typically develops in medium- to large-breed dogs, notably Golden and Labrador Retrievers, at a median age of 7–9 years, slightly younger than in the present study [5]. Moreover, the present study found that mixed breeds, Poodles, and Shih Tzus were most commonly diagnosed with fibrosarcoma, which is in contrast with a previous report that identified Golden Retrievers as the predominant breed [28].

Osteosarcoma development in humans and canines is influenced by common risk factors, including sex, growth, and puberty [29]. In humans, osteosarcoma often emerges during rapid bone growth in puberty, affecting taller individuals [29]. In canines, osteosarcoma tends to occur in large breeds during the time of late bone closure in canines, primarily affecting weight-bearing bones [30]. In the present study, osteosarcoma cases were found in the mandible, followed by the maxilla and the hard palate. Aggressive management, such as mandibulectomy and maxillectomy combined with neoadjuvant and/or adjuvant RT and chemotherapy, is indicated [31]. Subsequent research revealed that gene expressions in affected dogs that responded and did not respond to chemotherapy treatment showed a resemblance to their human counterparts [29]. This discovery highlights the potential significance of early diagnosis in ensuring successful treatment outcomes for canine oral osteosarcoma.

Canine acanthomatous ameloblastoma was the most frequent type of oral benign tumor in the present study. Previous studies have identified acanthomatous ameloblastoma as the most predominant odontogenic neoplasm in dogs, originating from diverse sources like basal epithelial cells of the oral mucosa, dental lamina, epithelial cell rests of Malassez, or the epithelial lining of an odontogenic cyst [32]. In this study, canine acanthomatous ameloblastoma was predominantly located in the gingiva and presented at a mean age of about 9 years; this finding is in agreement with previous research indicating that this tumor tends to occur in adult dogs [33,34]. Mixed breeds were overrepresented among dogs with canine acanthomatous ameloblastoma.

In the present study, the gingiva was the primary location for both benign and malignant oral tumors, followed by the lip, buccal mucosa, and tongue. The current results align with previous reports [4,6], underscoring the significance of regular oral examinations in dogs for timely tumor detection. Furthermore, the tongue and mandible were the second-most common sites for benign tumors, while the lip, buccal mucosa, and hard palate were the most common sites for malignant tumors. This information could be useful for veterinarians in identifying high-risk areas for tumor development, and veterinarians could target these areas during routine examinations. The present findings revealed that acanthomatous ameloblastoma was exclusively located in the gingiva, while melanoma and SCC were found in various oral sites, suggesting that different types of tumors may have distinct pathogeneses and require different diagnostic and treatment approaches. Moreover, the clinical staging according to the WHO staging system revealed that the most common stage for all malignant tumors in this study was stage III, with tumor diameters larger than 4 cm found frequently. This finding suggests that these tumors may have been diagnosed at a later stage, partly due to insufficient regular oral hygiene care and infrequent oral examinations.

In human medicine, the measurement of cell-free DNA (cfDNA) is extensively employed to monitor tumor characteristics and facilitate cancer treatment surveillance [35]. Notably, targeted sequencing studies have revealed that known driver mutations observed in human melanoma are infrequently encountered in canine melanoma, and investigations focused on cfDNA in canine melanoma remain scarce [36]. A recent study in veterinary medicine indicates that monitoring of long fragments of long interspersed nuclear element-1 from cfDNA and calculation of the DNA integrity index could serve as promising new biomarkers for monitoring the progression of oral malignant melanoma in dogs [16]. This development is a promising non-invasive tool for the diagnosis of oral neoplasms in dogs in the future.

There are several limitations to the present study. First, data were collected from a single participating referral center. This could have resulted in reduced external validity, meaning that the findings may not be generalizable to a broader population. Furthermore, the study’s sample size was limited, and there was missing data regarding survival time for patients with malignant tumors. In human medicine, oral cancer development involves various risk factors: tobacco smoking, alcohol consumption, and HPV are extensively studied factors, while inflammation and genetic susceptibility also play a crucial role [7]. Nonetheless, the present study did not investigate additional potential risk factors linked to the emergence of oral tumors, such as diet, environmental influences, and oral hygiene. Prospective studies with larger sample sizes and more comprehensive data collection methods are needed to further investigate the risk factors associated with oral tumors in dogs.

## Conclusion

The present study indicates a higher incidence of canine oral malignant tumors compared to canine oral benign tumors, with melanoma and SCC emerging as the predominant malignancies. Notably, the gingiva exhibited the highest frequency of both benign and malignant lesions. Moreover, the study highlights a heightened occurrence of oral tumors in senior and geriatric dogs and mixed-breed canines. These findings emphasize the increasing prevalence of oral tumors in dogs, providing valuable insights into their types and distribution, which can inform the development of more efficacious treatment strategies.

## List of abbreviations

cfDNA, cell-free DNA; CTLA-4, cytotoxic T-lymphocyte-associated antigen-4; RT, radiation therapy; SCC, squamous cell carcinoma; TNM, tumour node metastasis; WHO, World Health Organization
